# A Systematic Review and Meta-Analysis of the Association between the FV H1299R Variant and the Risk of Recurrent Pregnancy Loss

**DOI:** 10.3390/biology11111608

**Published:** 2022-11-03

**Authors:** Anna Paola Capra, Alessio Ardizzone, Silvana Briuglia, Maria Angela La Rosa, Stefania Mondello, Michela Campolo, Emanuela Esposito

**Affiliations:** 1 Department of Chemical, Biological, Pharmaceutical and Environmental Sciences, University of Messina, Viale Ferdinando Stagno D’Alcontres 31, 98166 Messina, Italy; 2 Department of Biomedical and Dental Sciences and Morphofunctional Imaging, University of Messina, Via Consolare Valeria 1, 98125 Messina, Italy; 3 Genetics and Pharmacogenetics Unit, “Gaetano Martino” University Hospital, Via Consolare Valeria 1, 98125 Messina, Italy

**Keywords:** recurrent pregnancy loss (RPL), H1299R, thrombophilia, thrombophilic gene variants, factor V (FV), miscarriages

## Abstract

**Simple Summary:**

Recurrent pregnancy loss (RPL) is a complex disorder affecting thousands of women around the world. The etiopathogenesis of RPL is not yet fully known and is considered to be multifactorial. Among the various causes, scientific research has identified the key role of thrombophilia. In this scenario, the H1299R variant in FV also seems to be involved; however, the relative data is often discordant. This study provides empirical evidence disproving the correlation between the FV H1299R variant and RPL, thus constituting a valid support for medical care during pregnancy and genetic counseling, in particular for gynecologists, obstetricians, and genetic counselors.

**Abstract:**

This study evaluated the association between the H1299R factor V (FV) variant (rs1800595) and recurrent pregnancy loss (RPL). Pubmed (MEDLINE) and Embase (OVID) bibliographic databases were searched from the inception to 31 May 2022 to identify suitable articles according to PRISMA and MOOSE guidelines. We included observational studies, case-control studies, cross-sectional studies, and cohort studies reporting a numerical and well-distinguished Het or Hom status of the H1299R variant obtained through PCR or other biochemical techniques and comparing RPL patients with a healthy control group. The review protocol was registered at PROSPERO (CRD42022330077). Two authors independently screened studies, extracted data, and carried out the risk of bias assessment using the Newcastle Ottawa scale (NOS). A meta-analysis was performed with RevMan software Version 5.4 using an odds ratio (OR) with an M-H, random effect, and 95% CI. We included 13 clinical studies for a total of 1669 RPL patients and 1466 healthy women as a control group. H1299R variant was slightly associated with RPL albeit without significance (OR 1.18, 95% CI: 0.78–1.80, *p* = 0.44). Subgroup analyses considering H1299R in heterozygosity (OR 1.13, 95% CI: 0.76–1.67, *p* = 0.56) and in homozygosity (OR: 2.11, 95% CI: 0.74–6.01, *p* = 0.16) revealed a similar trend. Lastly, we evaluated the association between H1299R and RPL based on the number of previous miscarriages (≥2 or ≥3). This comprehensive systematic review and meta-analysis sheds light on the specific influence of the H1299R variant in the F5 gene on RPL, constituting valid support for medical care during pregnancy and genetic counseling.

## 1. Introduction

Recurrent pregnancy loss (RPL) constitutes about 1% of all cases of pregnancy loss (PL) [1]. Unlike sporadic abortion, RPL is considered a condition in which a physical exam and testing are recommended as well as dedicated care and monitoring in future pregnancies.

Considering the emotional impact of this experience, both men and women affected by RPL show high risks of developing depression and anxiety, and they very often need psychological support to overcome the traumatic event [2].

Moreover, women with a history of PL showed an increased risk for subsequent loss [1].

There are different classifications of RPL; in fact, according to the European Society of Human Reproduction and Embryology (ESHRE) and the American Society for Reproductive Medicine (ASRM) guidelines, it is defined as the loss of 2 or more pregnancies before 24 weeks of pregnancy [3]. Conversely, 3 or more fetal losses before 24 weeks of pregnancy are considered as RPL by the Royal College of Obstetricians and Gynecologists (RCOG) [4].

Although multiple risk factors for RPL have been identified, the pathogenesis of RPL remains undetermined in more than 50% of cases [5].

It is important to pay attention to medical conditions present in the family or recurring in the reproductive history of the couples, recommending, in particular, to base the prognosis on the number of previous abortions and the female age [6]. As a multi-etiological complex disease, RPL involves the interaction of genetic factors and environmental factors. Known predisposing conditions include chromosomal and uterine anomalies, endometrial infections, endocrine disorders, autoimmune syndromes, and thrombophilic events.

Thrombophilia is a blood coagulation condition predisposing a hypercoagulability status. Thrombophilia can be inherited or acquired, and it is commonly associated with an increased risk of venous thromboembolism (VTE) [7]. Hereditary thrombophilia can be a contributing factor for pregnant women associated with an increased risk of RPL when compared with the wild-type group [4]. The risk of serious pregnancy complications, including embryonic/fetal loss, is probably linked to the tendency to form blood clots in the uterine environment and the alteration of the placental blood flow [8].

In a small group of patients, antithrombotic prophylaxis during pregnancy could reduce complications and increase the live birth rate [1,8].

Mutations in the *F5* gene (Coagulation Factor V) are among the most common causes of inherited thrombophilia.

In particular, the well-known Leiden mutation G1691A (Factor V Leiden, rs6025) causes APC resistance which is associated with an increased risk of VTE and pregnancy complications, such as RPL [4,9].

In 1996, another missense variant in exon 13 of the *F5* gene A4070G, p.(His1299Arg), known as R2 or H1299R (rs1800595), was identified and linked to hereditary thrombophilia. Previous studies have shown that His1299Arg change will increase the VTE risk 2–3 fold [9].

FV H1299R is associated with mild APC resistance and with a relative excess of the more thrombogenic isoform FV in plasma [10]. 

Recent findings indicate the H1299R variant in Factor V (FV) as an important influencing factor in the risk of fetal loss, intrauterine growth restriction (IUGR) and stillbirth, early miscarriage, and pre-eclampsia [10,11]. 

Nevertheless, conflicting data seems to disprove these assumptions, excluding the key role of H1299R in the etiopathogenesis of RPL [9].

Therefore, based on this unresolved question, this study aimed to perform an exhaustive meta-analysis in order to better understand the association between the FV variant H1299R and the risk of RPL.

## 2. Methods

### 2.1. Eligibility Criteria, Information Sources, and Search Strategy

The protocol of this study has been recorded in the PROSPERO register (CRD42022330077) and has already been published [12]. Systematic research followed the guidelines of Preferred Reporting Items for Systematic Reviews and Meta-analyses (PRISMA) and MOOSE.

APC and AA performed a bibliographic search using MEDLINE (PubMed) and EMBASE (Ovid) databases according to the eligibility criteria summarized in Table 1, and specific keywords as previously indicated [12].

Two content experts (SB and EE) designed a search strategy and supervised the study.

### 2.2. Study Selection

After removing duplicates from the two different databases, two review authors (APC and AA) individually screened the titles and abstracts of all records identified to remove articles that were not relevant. Next, we examined the full-text articles to select the records that corresponded most closely to the eligibility criteria; conflicting opinions were resolved through the mediation of a third review author (SB).

### 2.3. Data Extraction

The individual extraction of the data from the included articles was executed by two authors (APC and AA). We collected the following data: author(s), year of publication, study catchment area (i.e., geographic zone), study population (i.e., RPL or healthy individuals), study period, study design, the H1299R variant, and Het or Hom genotype as well as the number of previous miscarriages and mean age of women. Events or non-events were expressed as the number of patients with the H1299R variant (Het and/or Hom) in RPL or healthy females, respectively.

### 2.4. Assessment of Risk of Bias

The quality of the included studies reported in this meta-analysis was independently assessed by two reviewers (APC, AA) using the Newcastle Ottawa scale (NOS; see Supplementary Table S1).

Then, the study’s quality was classified as low, medium, or high on the basis of the NOS score. Disagreements in score assignments were resolved through discussion or the intervention of a third review author (SB).

### 2.5. Data Synthesis

For statistical analysis, we used an odds ratio (OR) measure and the random-effects model with the Mantel-Haenszel method. At the end, we obtained pooled estimates of the variant effect (OR) with the associated 95% confidence interval (CI).

The heterogeneity was originally evaluated through a graphical examination of the forest plots and then assessed using the I^2^ statistic as previously described [12]. Subgroup analyses were conducted to investigate the influence of the geographical area, mean age of RPL patients, and minimum previous pregnancies losses. Sensitivity analyses were conducted to assess the strength of the obtained outcomes and the impact of including studies without a high-risk of biased articles.

Review Manager (Rev Man. Version 5.4. Copenhagen: The Nordic Cochrane Centre, The Cochrane Collaboration, 2014) was used to perform the meta-analysis of the obtained data.

### 2.6. Preliminary Analyses

#### 2.6.1. Study Selection

In the first step, we identified 7391 records using the two databases PubMed (MEDLINE) and Embase (OVID). For this purpose, Medical Subject Headings (MeSH) referring to the thrombophilic variant H1299R and RPL were used as previously illustrated in the study protocol [12]. Five further records were obtained from additional sources.

After removing duplicates, we retrieved 3382 records to be screened for inclusion. We excluded 3347 articles after reading the title and abstract because they were not relevant to our review question.

Then, we examined the full-text of 35 articles for eligibility, excluding 22 records as they did not match the established inclusion and exclusion criteria.

**Figure 1:**
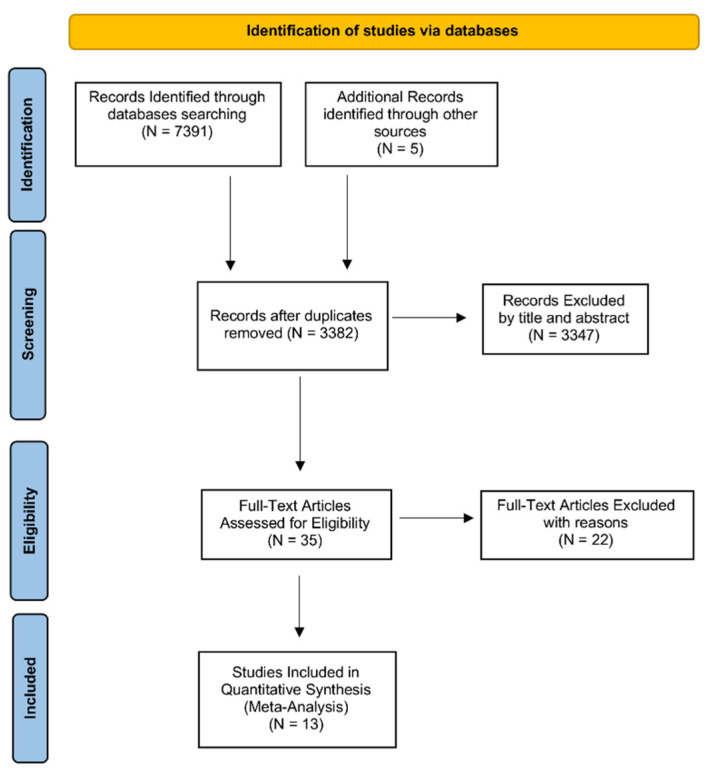
PRISMA Flow Diagram. The figure illustrates the PRISMA Flow Diagram for Search. The flow diagram was created according to PRISMA-P guidelines.

Finally, we included 13 studies in our systematic review and meta-analysis, as shown by the PRISMA Flowchart reported in Figure 1.

#### 2.6.2. Study Characteristics

We included 13 clinical studies for a total of 3135 women, of which 1669 RPL and 1466 healthy subjects. The main characteristics of the included studies are summarized in Table 2.

**Figure 2:**
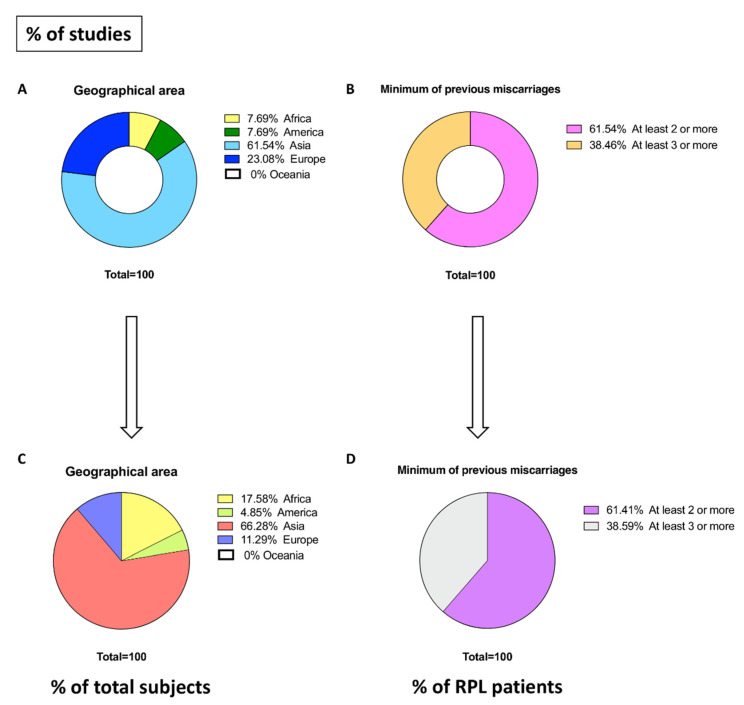
Preliminary analysis of the included studies. Charts on the geographical area distribution and number of previous miscarriages of the included studies. Charts describe: (**A**) the geographical area of included studies subdividing them into five macro areas; (**B**) the percentage of included studies reporting the minimum of previous miscarriages; (**C**) the percentage of subjects for every continent; (**D**) the percentage of RPL patients based on previous miscarriages.

After analysis of the data extracted from every single study, we carried out preliminary evaluations. Relatedly, of the 13 records included, 8 investigated the influence of H1299R in the Asian population (61.54%, Figure 2A) for a total of 2078 subjects (66.28%, Figure 2C), 3 were referable to the European population (23.08%, Figure 2A) for a total of 354 subjects (11.29%, Figure 2C), 1 was referrable for the African population (7.69%, Figure 2A) with 551 subjects (17.58%, Figure 2C) and 1 was referrable for the American population (7.69%, Figure 2A) with 152 subjects (4.85%, Figure 2C). No included records had targeted analyses of the Australian population (0%, Figure 2A,C). Of the 13 total records, 8 studies (61.54%, Figure 2B) examined RPL patients with at least 2 or more previous pregnancy losses (amount of RPL patients: 1025, 61.41% Figure 2D). Conversely, 5 studies (38.46%, Figure 2B) had taken into consideration RPL patients with at least 3 or more previous pregnancy losses (amount of RPL patients: 644, 38.59% Figure 2D).

#### 2.6.3. Risk of Bias of Included Studies

The NOS scores are shown in Supplementary Table S1.

After the authors’ evaluations, only three articles were considered to be at moderate risk of bias as they lacked specific data on the mean ± SD age of the individuals. 

For this reason, to further assess the validity of our study, we performed a sensitivity analysis excluding the studies with a low NOS score. 

Therefore, we excluded the studies of Arabkhazaeli et al., Dilley et al., and Torabi et al. in order to ensure they did not compromise the results of the entire survey and lead to misleading conclusions in our meta-analysis.

All the other articles were considered low risk, scoring well on the NOS scale.

## 3. Results

### 3.1. The Association between the H1299R Variant and RPL

**Figure 3:**
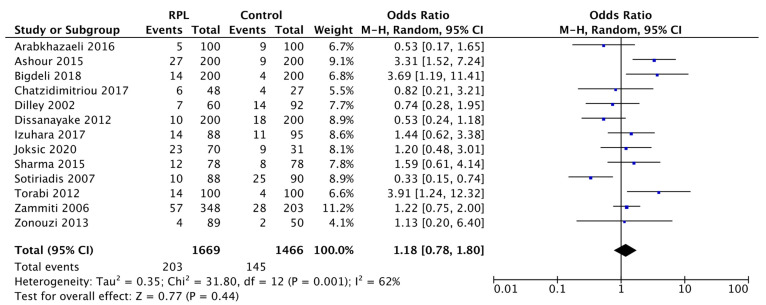
Forest plot of the association between H1299R and RPL. Squares display the effect estimate (ORs) with the size of each blue square corresponding to the weight given to each study in the meta-analysis. Horizontal lines represent the 95% CIs corresponding to each effect estimate. The black diamond represents the overall effect of intervention with its width representing the overall 95% CI. The I^2^ statistic represents a measure of heterogeneity. Overall effect OR: 1.18 [0.78, 1.80]; *p* = 0.44.

First, we evaluated the correlation between the H1299R variant and RPL not considering the heterozygous or homozygous status (Figure 3).

The 13 articles included showed an ORs range between 0.33–3.91. Despite the considerable degree of heterogeneity between studies (I^2^ = 62%), the total OR was 1.18 (95% CI: 0.78–1.80; test for overall effect *p* = 0.44).

This result, even if it did not reach statistical significance, indicated an 18% increase in the H1299R-RPL incidence compared to the control group.

To better estimate the influence of the H1299R variant on RPL, we separately evaluated the heterozygous AG and homozygous GG for A4070G.

**Figure 4:**
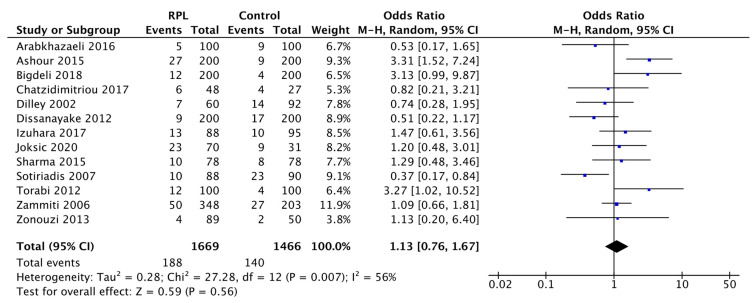
Forest plot of the association between H1299R heterozygous and RPL. The statistical value of every single study is expressed as OR. The horizontal black lines represent the confidence interval (95% CI). The blue squares indicate the weight of each study. The black diamond indicates the overall result of the statistical analysis. Heterogeneity is expressed as I^2^. Overall effect OR: 1.13 [0.76, 1.67]; *p* = 0.56.

In the analysis referring to the H1299R variant in heterozygosity, the 13 eligible revealed a range of ORs between 0.37–3.31 (Figure 4). In this assessment, the degree of heterogeneity was I^2^ = 56%. Considering the overall effect, the total OR was 1.13 (95% CI: 0.76–1.67, *p* = 0.56).

Thus, the statistical analysis of the H1299R variant in heterozygosity revealed an increase of 13% in RPL compared to the healthy female group. However, despite the fact that the trend was also comparable to the precedent in this case, the result was not significant (Test for overall effect *p* = 0.56).

**Figure 5:**
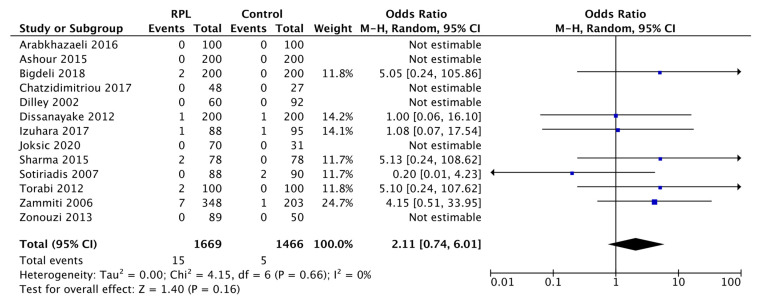
Forest plot of the association between H1299R homozygous and RPL. The forest plot illustrates the 95% CI for each study (black lines); the weight of each study (blue square) and the overall result (black diamond). Heterogeneity is expressed as I^2^ measure. Overall effect OR: 2.11 [0.74, 6.01]; *p* = 0.16.

The risk estimation of RPL associated with the H1299R variant in homozygous status is summarized in Figure 5.

In this forest plot, the ORs of the studies by Arabkhazaeli et al., 2016, Ashour et al., 2015, Chatzidimitriou et al., 2017, Dilley et al., 2002, Joksic et al., 2020, Zonouzi et al., 2013 were “Not estimable” as these studies did not find the presence of homozygous H1299R subjects in either the RPL or control group.

The ORs of the estimated studies ranged from 0.20 to 5.13. The analysis of pooled data from the 13 studies indicated a noticeable association between the H1299R variant in homozygosity and RPL (OR: 2.11, 95% CI: 0.74–6.01), albeit not significantly (*p* = 0.16). Indeed, with an OR of 2.11, the presence of GG status for the A4070G variant increased in RPL individuals by 111% compared to the successful pregnancy group. The degree of statistical heterogeneity was I^2^ = 0%.

### 3.2. Additional Analyses

As indicated by the general recommendations for RPL cases, the number of previous miscarriages is critical information for the prognosis of patients [6].

**Figure 6:**
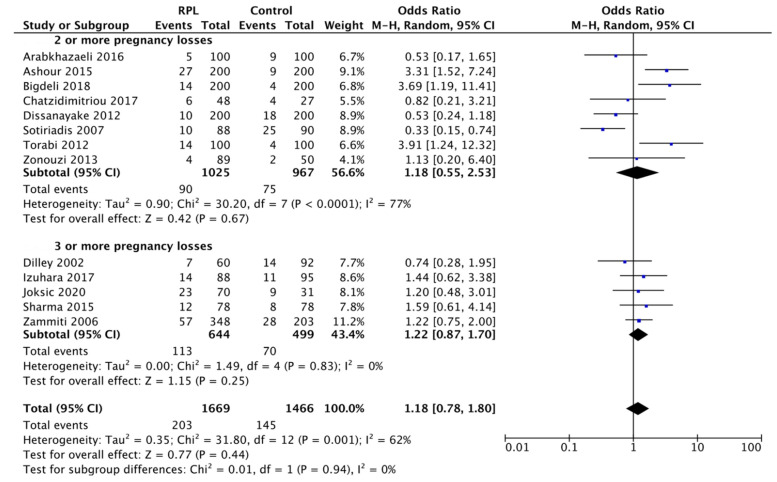
Subgroup analysis of the H1299R and RPL according to minimum previous pregnancy losses. The results of the meta-analysis are expressed as ORs. Black horizontal lines represent the 95% CIs of the single study. The weight given to each study is revealed by the size of the blue square. The black diamond represents the overall effect of the intervention. The statistical value I^2^ indicates the heterogeneity. In the subgroup analysis the number of events indicates the presence of H1299R variant without distinction between Het and Hom. 2 or more pregnancy losses (OR: 1.18 [0.55, 2.53]; *p* = 0.67); 3 or more pregnancy losses (OR: 1.22 [0.87, 1.70]; *p* = 0.25). Overall effect OR: 1.18 [0.78, 1.80]; *p* = 0.44. Test for subgroup differences (*p* = 0.94).

Thus, we performed a subgroup analysis between studies indicating RPL as 2 or more pregnancy losses according to ESHRE and ASRM recommendations or studies indicating RPL as 3 or more pregnancy losses based on RCOG advice (Figure 6). Analysis of the first subgroup showed 1.18 OR, 95% CI: 0.55–2.53, *p* = 0.67. Similar data was also found in the second subgroup 1.22 OR, 95% CI: 0.87–1.70, *p* = 0.25. Statistical significance was not observed in either group. In light of this, it is possible to state that the different definitions of RPL reported by the ESHRE/ASRM or RCOG guidelines did not affect the result of this study.

**Figure 7:**
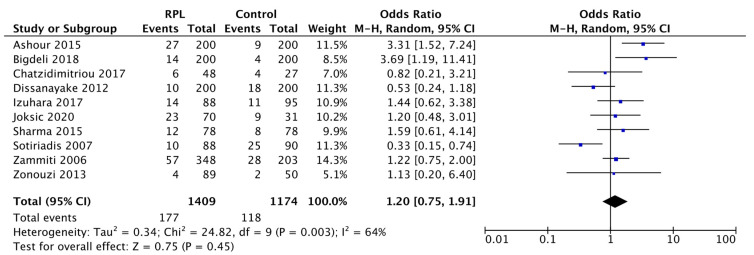
Sensitivity analysis of the H1299R and RPL by excluding NOS low score studies. The forest plot illustrates the horizontal black lines representing the 95% CIs and the weight of each study as a blue square. The black diamond represents the estimate of the overall effect. I^2^ is an index of heterogeneity. In the sensitivity analysis, the number of events indicates the presence of the H1299R variant without distinction between Het and Hom. Overall effect OR: 1.20 [0.75, 1.91]; *p* = 0.45.

Moreover, as shown in Figure 7, the pooled data of the sensitivity analysis denoted an OR of 1.20 (95% CI: 0.75–1.91; *p* = 0.45). These outcomes are comparable to those shown in the plot of Figure 3 (OR: 1.18 [0.78, 1.80], *p* = 0.44), in which all eligible studies were included. This proved that the inclusion of the three high-risk bias studies (with the lowest NOS scale score) did not compromise the results of our meta-analysis.

## 4. Discussion

At present, RPL is a heterogeneous condition caused by environmental and genetic factors. The etiology of RPL cases remains undefined, and a limit to estimating the risk of recurrence persists in clinical practice [24].

Hormonal and physical changes in pregnancy increase the possible formation of clots in the placental blood vessels, thus leading to RPL [25,26]. 

To avoid clinical complications, drug treatment may be prescribed according to the individual clinical history; usually, this is based on the administration of acetylsalicylic acid as an antiaggregant and/or heparin as an anticoagulant [27]. In fact, it has been demonstrated how the modulation of the coagulation cascade might protect pregnancies by reducing the binding of antiphospholipid antibodies and inflammation, also facilitating implantation and inhibiting complement activation [28].

In the last few decades, the attention of researchers has been directed to Factor V, a blood-clotting protein, also known as proaccelerin or labile factor [29]. Unlike most other clotting factors, FV is not enzymatically active but functions as a cofactor of Factor X to activate the prothrombin into thrombin, which in turn allows the formation of fibrin and, therefore, of the clot starting from the fibrinogen molecule [30]. Some of the Factor V variants, such as the variant of Leiden G1691A, have been identified as the most common genetic risk factor for hereditary thrombophilia, even for RPL patients [31]. 

In addition to the FV Leiden mutation, another variant, commonly referred to as H1299R, affects the plasma concentration of FV and causes a mild resistance to activated protein C [32].

This missense variant, the substitution of a nucleotide residue A 4070 in G in the *F5* gene, results in the change of codon 1299 Histidine into a different amino acid Arginine and has also been described as polymorphism R2 [33]. The H1299R variant has been associated with an increased risk of thrombosis alone or in compound heterozygosity with the FV Leiden mutation [34]. The presence of the H1299R variant in FV would increase the risk of RPL [11]; on the other hand, the high variability of the results regarding the association between this polymorphism and the risk of miscarriage does not support clear conclusions. [9].

Our meta-analysis included 13 studies with a total of 3135 subjects: 1466 healthy women as a control group and 1669 RPL patients. Compared to the reference group, our evidence suggested that the H1299R variant was not significantly associated with RPL, although the trend showed a slight increase in the RPL-related incidence of the H1299R. More precisely, in our first assessment without distinction between Het or Hom status, pooled data indicated an increase of 18% in the H1299R variant for the RPL subjects (OR: 1.18, 95% CI: 0.78–1.80, Test for overall effect *p* = 0.44).

Since the rarity of homozygotes and the more frequent heterozygous condition have a greater public health impact [35], we estimated heterozygous AG and homozygous GG for A4070G to make a more careful evaluation. The presence of the H1299R variant in heterozygosity was increased by 13% in the RPL group compared to the reference group (pooled data OR 1.13, 95% CI: 0.76–1.67, *p* = 0.56). Likewise, the H1299R variant in homozygosity was related to the RPL group to a greater extent than the healthy group by 111%, although not in a significant way (pooled data OR: 2.11, 95% CI: 0.74–6.01, *p* = 0.16). This pooled data is influenced by the studies by Dissanayake et al., Izuhara et al., and Sotiriadis et al., in which the presence of GG subjects was equal in both groups or superior in the control group. Moreover, the lower frequency of homozygous variants compared to heterozygotes should be considered, which results in limited evidence for RPL H1299R homozygous women with no clear outcome.

Alternatively, when a subgroup analysis was carried out considering the different criteria for defining RPL in current guidelines, a comparable trend emerges for both the ESHRE and ASRM definition (pooled data: 1.18 OR, 95% CI: 0.55–2.53, *p* = 0.67) or RCOG (pooled data: 1.22 OR, 95% CI: 0.87–1.70, *p* = 0.25). 

The relation between the H1299R variant and RPL, as reported, was also negligible in the sensitivity analysis (pooled data: 1.20 OR, 95% CI: 0.75–1.91, *p* = 0.45).

Considering the limited number of included records (13 articles) and the high degree of heterogeneity between the studies, the result should be interpreted with caution. 

The statistics of genotypes and allelic frequencies, excluding the weight of the individual studies as well as the degree of heterogeneity, indicate a similar trend but with a considerable significance (see Supplementary Tables S2 and S3). 

Other limitations of this meta-analysis must be addressed. Some important factors could play a role, such as age, co-morbidities status, smoking habit, previous at-term pregnancy, body mass index (BMI), or other genetic variants involved. In addition, we cannot exclude a lower prevalence of chromosomal abnormalities, autoimmune and thyroid disorders, or other conditions not diagnosed or not reported in the study groups but are associated with an increased risk of RPL.

Unfortunately, karyotype analysis was not present in all the selected articles, and this is well-known contributing information to the individual risk assessment of RPL women. However, what remains crucial is that genetic counseling and information on care and treatment options should be offered to RPL couples [6].

No systematic reviews and meta-analyses have specifically addressed the role of H1299R in the context of recurrent miscarriage. The most recent scientific evidence, given by Liu et al. [36], reports the role of hereditary thrombophilia and recurrent pregnancy loss but does not exclusively focus on H1299R.

## 5. Conclusions and Limitations

This meta-analysis demonstrated that there is no clear association between the FV H1299R variant and RPL. 

The present study lays a basis for future research in the H1299R variant and RPL, constituting valid support for medical care during pregnancy and genetic counseling.

However, the limitations of this meta-analysis must be addressed. First, the sample size was relatively small and might not have provided sufficient power to estimate the associations. 

Hence, large studies on RPL couples would provide significant new insights into the clinical relevance of genetic tests and other specific marker evaluations and should also consider the male contribution more deeply.

## Figures and Tables

**Table 1:** Description of inclusion and exclusion criteria.

Inclusion Criteria	Exclusion Criteria
Observational studies, case-control studies, cross-sectional studies and cohort studies	Editorials, letters, reviews, guidelines, case reports, abstracts and paper conferences, systematic reviews and meta-analyses, and ongoing studies
Articles written in English	Studies that enrolled male subjects as control groups
Articles reporting a numerical and well-distinguished Het or Hom status of H1299R variant	Studies using literature data as control groups
Articles describing RPL patients with at least two or more previous miscarriages compared to a control group having at least one full-term pregnancy.	Studies on couples

**Table 2:** Descriptions of eligible studies.

First Author and Year of Publication	Title of the Study	Patients (RPL:Control)	Mean Age of RPL Patients	Reference
Arabkhazaeli et al., 2016	H1299R in coagulation Factor V and Glu429Ala in MTHFR genes in recurrent pregnancy loss in Sari, Mazandaran	100:100	~32.5	[9]
Ashour et al., 2015	The relationship between gene polymorphisms of coagulation factors II, V and XI and risk of recurrent pregnancy loss in Palestine	200:200	33.2 ± 5.4	[13]
Bigdeli et al., 2018	Association between thrombophilia gene polymorphisms and recurrent pregnancy loss risk in the Iranian population	200:200	23.0 ± 3.8	[11]
Chatzidimitriou et al., 2017	Thrombophilic gene polymorphisms and recurrent pregnancy loss in Greek women	48:27	35.3 ± 5.08	[14]
Dilley et al., 2002	Mutations in the factor V, prothrombin and MTHFR genes are not risk factors for recurrent fetal loss	60:92	~33.5	[15]
Dissanayake et al., 2012	Candidate gene study of genetic thrombophilic polymorphisms in pre-eclampsia and recurrent pregnancy loss in Sinhalese women	200:200	32.1 ± 5.6	[16]
Izuhara et al., 2017	Genotyping analysis of the factor V Nara mutation, Hong Kong mutation, and 16 single-nucleotide polymorphisms, including the R2 haplotype, and the involvement of factor V activity in patients with recurrent miscarriage	88:95	33.0 ± 4.2	[17]
Joksic et al., 2020	Combined presence of coagulation factor XIII V34L and plasminogen activator inhibitor 1 4G/5G gene polymorphisms significantly contribute to recurrent pregnancy loss in Serbian population	70:31	33.2 ± 5.4	[18]
Sharma et al., 2015	Polymorphisms in factor V and antithrombin III gene in recurrent pregnancy loss: a case–control study in Indian population	78:78	28.6 ± 3.32	[19]
Sotiriadis et al., 2007	Combined Thrombophilic Mutations in Women with Unexplained Recurrent Miscarriage	88:90	32.2 ± 4.7	[20]
Torabi et al., 2012	Combination of thrombophilic gene polymorphisms as a cause of increased the risk of recurrent pregnancy loss	100:100	Not specified	[21]
Zammiti et al., 2006	Association of factor V gene polymorphisms (Leiden; Cambridge; Hong Kong and HR2 haplotype) with recurrent idiopathic pregnancy loss in Tunisia	348:203	28.93 ± 5.92	[22]
Zonouzi et al., 2013	The association between thrombophilic gene mutations and recurrent pregnancy loss	89:50	30.18 ± 4.95	[23]

## Data Availability

Data of this study are available to the corresponding author’s address.

## Supplementary Materials

The following supporting information can be downloaded at: https://www.mdpi.com/article/10.3390/biology11111608/s1. Table S1: Table with Risk of Bias judgement according NOS score, Table S2: Table summarizing genotypes and allele frequencies for RPL and Control group respectively, Table S3: Table summarizing statics for total genotypes and allele frequencies in RPL and Control group respectively

## Abbreviations

PL	Pregnancy Loss
RPL	Recurrent Pregnancy Loss
ESHRE	European Society of Human Reproduction and Embryology
ASRM	American Society for Reproductive Medicine
RCOG	Royal College of Obstetricians and Gynecologists
VTE	Venous thromboembolism
PRISMA	Preferred Reporting Items for Systematic Reviews and Meta-analyses
Het	Heterozygosity Hom Homozygosity
NOS	Newcastle Ottawa scale
OR	Odds Ratio
CI	Confidence interval
MeSH	Medical Subject Headings
BMI	Body mass index
