# Supplementary material for: A Systematic Review and Meta-Analysis of the Association between the FV H1299R Variant and the Risk of Recurrent Pregnancy Loss
Source: Biology (Basel). 2022 Nov 3;11(11):1608. doi: 10.3390/biology11111608 (PMC9687207; doi:10.3390/biology11111608)
Supplement: Supplementary file 1 [file biology-11-01608-s001.zip › Supplementary Table 1.pdf]

RISK OF BIAS Table	SELECTION				COMPARABILITY		OUTCOME			
	Representative of the exposed cohort	Selection of external control	Ascertainment of exposure	Outcome of interest not present at the start of the study	Comparability of Cohorts		Assessment of outcomes	Sufficient follow-up time	Adequacy of follow-up	
					Main Factor	Additional Factor				
	1	2	3	4	5	6	7	8	9	OVERALL
Arabkhazaeli et al. 2016	x	x			x		x			4
Ashour et al. 2015	x	x	x		x	x	x			6
Bigdeli et al. 2018	x	x	x		x	x	x			6
Chatzidimitriou et al. 2017	x	x	x		x	x	x			6
Dilley et al. 2002	x	x			x		x			4
Dissanayake et al. 2012	x	x	x		x	x	x			6
Izuhara et al. 2017	x	x	x		x	x	x			6
Joksic et al. 2020	x	x	x		x	x	x			6
Sharma et al. 2015	x	x	x		x	x	x			6
Sotiriadis et al. 2007	x	x	x		x	x	x			6
Torabi et al. 2012	x	x			x		x			4
Zammiti et al. 2006	x	x	x		x	x	x			6
Zonouzi et al. 2013	x	x	x		x	x	x			6

Supplementary Table S1. Table with Risk of Bias judgement according NOS score
