# Supplementary material for: A Systematic Review and Meta-Analysis of the Association between the FV H1299R Variant and the Risk of Recurrent Pregnancy Loss
Source: Biology (Basel). 2022 Nov 3;11(11):1608. doi: 10.3390/biology11111608 (PMC9687207; doi:10.3390/biology11111608)
Supplement: Supplementary file 1 [file biology-11-01608-s001.zip › Supplementary Table 2.pdf]

First author	year	RPL					Sample size^{RPL}	Frequenze alleliche A^{RPL}	Frequenze alleliche G^{RPL}	RPL			HWE		Control					Sample size^{CTR}	Frequenze alleliche A^{CTR}	Frequenze alleliche G^{CTR}	MAF
		AA	AG	GG	A	G				Attese AA	Attese AG	Attese GG	x2	*P*	AA	AG	GG	A	G				
**Arabkhazaeli et al.**	2016	95	5	0	195	5	100	0,975	0,025	95,0625	4,875	0,0625	0,065746	0,967661	91	9	0	191	9	100	0,96	0,045	0,045
**Ashour et al.**	2015	173	27	0	373	27	200	0,933	0,068	173,911	25,178	0,911	1,048	0,592	191	9	0	391	9	200	0,98	0,0225	0,023
**Bigdeli et al.**	2018	186	12	2	384	16	200	0,960	0,040	184,320	15,360	0,320	9,570	0,008	196	4	0	396	4	200	0,99	0,01	0,010
**Chatzidimitriou et al.**	2017	42	6	0	90	6	48	0,938	0,063	42,188	5,625	0,188	0,213	0,899	23	4	0	50	4	27	0,93	0,074074074	0,074
**Dilley et al.**	2002	53	7	0	113	7	60	0,942	0,058	53,204	6,592	0,204	0,230	0,891	78	14	0	170	14	92	0,92	0,076086957	0,076
**Dissanayake et al.**	2012	190	9	1	389	11	200	0,973	0,028	189,151	10,698	0,151	5,036	0,081	182	17	1	381	19	200	0,95	0,0475	0,048
**Izuhara et al.**	2017	74	13	1	161	15	88	0,915	0,085	73,639	13,722	0,639	0,243	0,885	84	10	1	178	12	95	0,94	0,063157895	0,063
**Joksic et al.**	2020	47	23	0	117	23	70	0,836	0,164	48,889	19,221	1,889	2,705	0,259	22	9	0	53	9	31	0,85	0,14516129	0,145
**Sharma et al.**	2015	66	10	2	142	14	78	0,910	0,090	64,628	12,744	0,628	3,615	0,164	70	8	0	148	8	78	0,95	0,051282051	0,051
**Sotiriadis et al.**	2007	78	10	0	166	10	88	0,943	0,057	78,284	9,432	0,284	0,319	0,852	65	23	2	153	27	90	0,85	0,15	0,150
**Torabi et al.**	2012	86	12	2	184	16	100	0,920	0,080	84,640	14,720	0,640	3,414	0,181	96	4	0	196	4	100	0,98	0,02	0,020
**Zammiti et al.**	2006	291	50	7	632	64	348	0,908	0,092	286,943	58,115	2,943	6,785	0,034	175	27	1	377	29	203	0,93	0,071428571	0,071
**Zonouzi et al.**	2013	85	4	0	174	4	89	0,978	0,022	85,0449438	3,91011236	0,044944	0,047034	0,976757	48	2	0	98	2	50	0,98	0,02	0,020
		**1466**	**188**	**15**	**3120**	**218**	**1669**	**0,935**	**0,065**						**1321**	**140**	**5**	**2782**	**150**	**1466**	**0,95**	**0,05**	

Supplementary Table S2. Table summarizing genotypes and allele frequencies for RPL and Control group respectively
