# Supplementary material for: A Systematic Review and Meta-Analysis of the Association between the FV H1299R Variant and the Risk of Recurrent Pregnancy Loss
Source: Biology (Basel). 2022 Nov 3;11(11):1608. doi: 10.3390/biology11111608 (PMC9687207; doi:10.3390/biology11111608)
Supplement: Supplementary file 1 [file biology-11-01608-s001.zip › Supplementary Table 3.pdf]

	Genotypes			Statistic		Alleles		Statistic	
Study Groups	**AA**	**AG**	**GG**	**X^2**	**P-Value**	**A**	**G**	**X^2**	**P-Value**
1669 RPL patients	1466	188	15	**6.45**	**0.039**	3120	218	**5.65**	**0.017**
1466 Control subjects	1321	140	5			2782	150		

Supplementary Table S3. Table summarizing statics for total genotypes and allele frequencies in RPL and Control group respectively
